# Selenium-Binding Protein 1 (SELENBP1) Supports Hydrogen Sulfide Biosynthesis and Adipogenesis

**DOI:** 10.3390/antiox10030361

**Published:** 2021-02-27

**Authors:** Elisa B. Randi, Giovanna Casili, Simona Jacquemai, Csaba Szabo

**Affiliations:** Chair of Pharmacology, Section of Medicine, University of Fribourg, CH-1700 Fribourg, Switzerland; giovanna.casili@unifr.ch (G.C.); simona.jacquemai@unifr.ch (S.J.)

**Keywords:** fat, obesity, gasotransmitters, mitochondria, differentiation, metabolism

## Abstract

Hydrogen sulfide (H_2_S), a mammalian gasotransmitter, is involved in the regulation of a variety of fundamental processes including intracellular signaling, cellular bioenergetics, cell proliferation, and cell differentiation. Cystathionine γ-lyase (CSE), cystathionine β-synthase (CBS), and 3-mercaptopyruvate sulfurtransferase (3-MST) are currently considered the three principal mammalian H_2_S-generating enzymes. However, recently, a fourth H_2_S-producing enzyme, selenium-binding-protein 1 (SELENBP1), has also been identified. The cellular regulatory role(s) of SELENBP1 are incompletely understood. The current study investigated whether SELENBP1 plays a role in the regulation of adipocyte differentiation in vitro. 3T3-L1 preadipocytes with or without SELENBP1 knock-down were subjected to differentiation-inducing conditions, and H_2_S production, cellular lipid accumulation, cell proliferation, and mitochondrial activity were quantified. Adipocyte differentiation was associated with an upregulation of H_2_S biosynthesis. SELENBP1 silencing decreased cellular H_2_S levels, suppressed the expression of the three “classical” H_2_S-producing enzymes (CBS, CSE, and 3-MST) and significantly suppressed adipocyte differentiation. Treatment of SELENBP1 knock-down cells with the H_2_S donor GYY4137 partially restored lipid accumulation, increased cellular H_2_S levels, and exerted a bell-shaped effect on cellular bioenergetics (enhancement at 1 and 3 mM, and inhibition at 6 mM). We conclude that SELENBP1 in adipocytes (1) contributes to H_2_S biosynthesis and (2) acts as an endogenous stimulator of adipocyte differentiation.

## 1. Introduction

Over the last two decades, the gaseous mediator hydrogen sulfide (H_2_S) has emerged as a mammalian, diffusible mediator, with important roles in the regulation of fundamental cellular functions in health and disease. The emergence of H_2_S as an endogenous mammalian mediator has been the subject of specialized review articles [1,2]. In addition, the various biological roles of H_2_S in the regulation of the cardiovascular, nervous, and immune systems, as well as the molecular biology, biochemistry, and pharmacology of the principal various H_2_S-producing enzymes has been summarized in specialized reviews [3,4,5,6,7,8,9,10,11,12,13,14,15,16,17,18,19,20]. H_2_S biosynthesis in mammalian cells is generally attributed to three enzymes: cystathionine-β-synthase (CBS), cystathionine-γ-lyase (CSE), and 3-mercaptopyruvate sulfurtransferase (3-MST); non-enzymatic reactions are also known to contribute. All of the above enzymes, directly or indirectly, utilize sulfur-containing amino acids as their substrates. H_2_S generated by these enzymes acts as a labile, diffusible mediator. It reaches various cellular compartments; it can also exit the cell to exert autocrine and paracrine biological actions. H_2_S can react with other labile diffusible species to create various secondary and tertiary species, with distinct biological roles; in a broader context, H_2_S is considered an essential component of the “reactive species interactome” (a collective term that also includes nitric oxide and various reactive oxygen species) [21,22,23]. 

Recent work has identified several, additional (alternative) enzymatic sources of H_2_S in mammalian cells and tissues. H_2_S can be generated from D-amino acid oxidase (DAO) and 3-MST in the kidney and gut [24,25], H_2_S (as well as polysulfides, i.e., closely related, diffusible sulfur species) can also be produced from L-cysteine by cysteinyl-tRNA synthetases (CARSs) [26]. Moreover, it has been recently suggested that H_2_S (together with H_2_O_2_) can also be produced by selenium-binding protein 1 (EC 1.8.3.4, abbreviated in the literature as SBP1, hSP56, or SELENBP1) [27,28]. SELENBP1 shares a 26% identity and a 54% sequence similarity with the mtoX gene from methylotrophic bacteria, indicating SELENBP1 as a human methanethiol oxidase (MTO) [27]. MTOs regulate the conversion of organically bound sulfur to inorganic sulfur, by transforming methanethiol into H_2_O_2_, formaldehyde, and H_2_S [27]. Methanethiol can be generated from (i) methionine by l-methionine γ-lyase or (ii) hydrogen sulfide (H_2_S) by S-methyltransferase [29]. The potential regulation and cellular function by these more recently identified biological H_2_S sources is incompletely understood.

## 2. Materials and Methods

### 2.1. Reagents

7-Azido-4-methylcoumarin (AzMC) was purchased from Sigma Aldrich (St Louis, MI, USA; Ref. 802409); its stock solution was made in DMSO (100 mM). Hank’s Balanced Salt Solution (HBSS) was purchased from Thermo Fisher Scientific (Waltham, MA, USA; Ref. 14170112). Red Oil was purchased from Sigma-Aldrich (Ref. O-0625); its stock solution was made by dissolving 0.7 g of Oil Red O powder in 200 ml isopropanol. The Oil Red O working solution was freshly prepared by mixing 6 parts of Oil Red O stock solution and 4 parts of dH_2_O and was then this solution was filtered with a 0.2 μm filter. Cell Proliferation Kit II (XTT, sodium 3´-[1-(phenylaminocarbonyl)-3,4-tetrazolium]-bis (4-methoxy-6-nitro) benzene sulfonic acid hydrate, Ref. 11465015001) for viability assay and Cell Proliferation ELISA BrdU (5-bromo-2’-deoxyuridine, Ref. 11647229001) were purchased from Roche. The H_2_S donor GYY4137 (Sigma-Aldrich, St Louis, MI, USA;) was dissolved in cell medium immediately before use.

### 2.2. Cell Culture, Cell Differentiation, and Pharmacological Treatments

The mouse fibroblast cell line 3T3-L1 MBX was purchased from ATCC^®^. Cells were cultured in Advanced DMEM/F12 (Thermo Fisher Scientific Waltham, MA, USA; Ref. 12634010) supplemented with 10% fetal bovine serum, 100 U/mL penicillin, and 100 µg/ml streptomycin. Cells were grown and maintained in flasks and were not allowed to reach confluence. For individual experiments, cells were seeded onto 6-well or 96-well plates at low confluence. To induce adipocyte differentiation (using a protocol modified from [30]), on Day 0 cells were treated with 1 µg/mL insulin and 1 µg/mL dexamethasone (“Differentiation Medium”). After 48 h, the medium was switched to one containing 1 µg/ml insulin (“Maintenance Medium”), which was renewed every 2 days. Undifferentiated cells (“Day 0 Controls”) were processed for experiments directly on Day 0. All experiments were performed on non-differentiated and differentiated cells, corresponding to Day 0 and Day 7 of the experimental protocol, respectively. Treatment with GYY4137 was performed at D0 and D4, during the differentiation process.

### 2.3. Generation of Stable Knock-Down Cell Lines

Stable SELENBP1 knock-down cell lines were generated as previously described [31]. Short hairpin RNA (shRNA) sequences targeting mouse SELENBP1 in a pLKO.1-puro plasmid were purchased from Sigma-Aldrich (sh85: order number TRCN0000101185; sh86: order number TRCN0000101186). Control cells (shCTR) were transfected with a non-targeting control shRNA (Sigma-Aldrich, St Louis, MO, USA). Cell selection was performed using puromycin.

### 2.4. Western Blotting

Cells were seeded in 6-well plates at 10^5^ cells/well. On the day of the experiment, cell medium was removed, cells were washed with PBS, and PathScan sandwich ELISA lysis buffer (Cell Signaling Technology, Danvers, MA, USA) supplemented with 1X Protease/Phosphatase Inhibitor Cocktail (Cell Signaling Technology) was added. After centrifugation, protein content of the lysates was quantified using the Bradford assay. For this, 20 µg of total protein was loaded onto 4–12% bis-tris gel and transferred to a nitrocellulose membrane with iBolt2 apparatus. Membranes were blocked for 1 h at room temperature and then incubated with specific primary antibodies overnight at 4 °C. The antibodies used in this study were: rabbit anti-SELENBP1 (Abcam, 1:1000), rabbit anti-Adiponectin (Sigma, 1:500), anti-CBS (Cell Signaling Technology, 1:1000), rabbit anti-CSE (Abcam 1:500), rabbit anti 3-MST (Abcam, 1:300), and mouse anti-β-Actin (Cell Signaling Technology, 1:5000). The latter was used as a loading control. Detection was performed with ECL Prime solution (Sigma-Aldrich, St Louis, MO, USA) using the Azure 600 analyzer (Azure biosystems).

### 2.5. Cell Proliferation Assay

Cells were seeded in 96-well plates at 5 × 10^3^ cells/well and cell proliferation was quantified as described [32]. Briefly, 20 μL of diluted BrdU solution were added to the cells and then the cells were incubated for 2 h at 37 °C in the dark. Cells were then fixed with a Fixdenat solution for 30 min and subsequently incubated with anti-BrdU-peroxidase antibody solution for 90 min at room temperature. Cells were washed three times with PBS and the substrate solution was added. Absorbance at 370 nm and the reference wavelength at 492 nm were analyzed using an Infinite M200 Pro plate reader (Tecan, Männedof, Switzerland). This assay measures the incorporation of BrdU in place of thymidine in newly synthetized DNA as a parameter for cellular proliferation. The incubation with the Fixdenat solution not only fixes the cells, but it denatures the DNA, thus allowing the binding of the antibody to the newly incorporated BrdU.

### 2.6. Mitochondrial Activity Assay

Cells were seeded in transparent 96-well plates at 5 × 10^4^ cells/well and subjected to an XTT-based assay that quantified the ability of the cells to convert XTT to formazan by mitochondrial reductases (i.e., a process that relies on the activity/integrity of the mitochondria) [33]. On the day of the experiment, 5 mL XTT labeling reagent was mixed with 0.1 mL electron coupling reagent and added to the cells (50 µL/well). After 2 h of incubation at 37 °C in the dark, the absorbance of the formazan at 490 nm and the reference wavelength at 680 nm were obtained using an Infinite M200 Pro plate reader (Tecan). This method measures the metabolic activity in the cells. XTT is cleaved to formazan by mitochondria and the detected color intensity correlates with the cells’ mitochondrial activity. 

### 2.7. Extracellular Flux Analysis

The Seahorse XFe-24 flux Analyzer (Agilent Technologies, Santa Clara, CA, USA) was used to measure mitochondrial respiration (O_2_ consumption), as previously described [32]. Cells were seeded on Seahorse XFe-24 Cell Culture Microplates at 2.5 × 10^3^ cells/well, differentiated, and eventually treated with GYY4137 as described above. On the day of the experiment, cell medium was replaced with Seahorse XF DMEM, supplemented with 20 µM glutamine, 10 µM pyruvate, and 1 mM glucose (final concentration), and bioenergetics parameters were calculated using the Agilent Seahorse Wave Desktop software (Agilent Technologies). 

### 2.8. Live Cell H_2_S Detection Using a Fluorescent Probe

H_2_S production was measured using AzMC, a H_2_S-selective fluorogenic probe as previously described [34,35]. In the presence of H_2_S, AzMC is reduced to the fluorescent AMC (7-amino-4-methylcoumarin). Cells were seeded in black 96-well plates at 5 × 10^3^ cells/well. AzMC stock was diluted in HBSS to a final concentration of 100 μM. After removing the cell medium, 100 μL of AzMC-HBSS solution was added to the cells, followed by a 1 h incubation at 37 °C in the dark. The AMC fluorescence intensity was quantified using an Infinite M200 Pro plate reader (Tecan) (excitation = 365 nm, emission = 450 nm).

### 2.9. Detection of Cellular Lipid Accumulation with Oil Red O Staining

Oil Red O, a hydrophobic soluble dye, was used for the cellular staining of triglycerides, fatty acids, and lipoproteins, as previously described [30]. Cells were seeded in 24-well plates at 2.5 × 10^4^ cells/well. On the day of the experiment, the medium was removed, and the cells were fixed using a solution of 4% paraformaldehyde in PBS. After 30 min at room temperature, cells were washed with 60% isopropanol, dried, and then stained with the Oil Red O working solution for 10 min. Subsequently, cells were washed several times with water and eventually pictures were taken. The plate was then dried, and the staining was eluted with 100% isopropanol. The absorbance of the dissolved Oil red O was measured with an Infinite M200 Pro plate reader (Tecan).

### 2.10. Statistical Analysis

Data are presented as representative blots or mean ± SEM values of experiments performed on at least *n* = 3 experimental days. ANOVA followed by post-hoc analysis by Dunnett’s multiple comparisons test were used to analyze the numerical data. A *p* < 0.05 was considered statistically significant.

## 3. Results

### 3.1. Effect of SELENBP1 Knock-Down on Suppression of Adiponectin Expression

**Figure 1:**
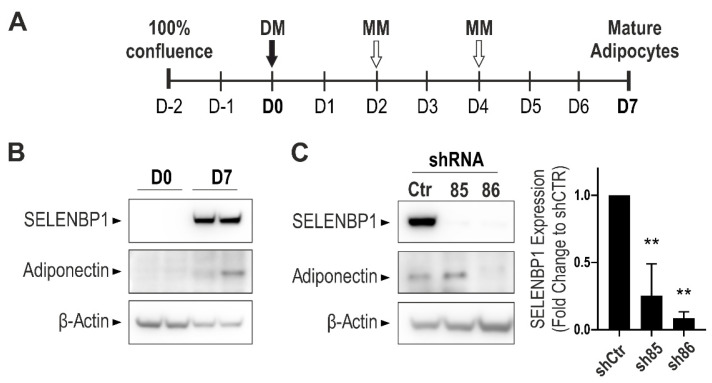
Assessment of the adipogenesis process in wild-type 3T3-L1 and shSELENBP1 cells. (**A**) Schematic overview of the differentiation process in 3T3-L1 cells. (**B**) Representative immunoblot showing the expression of SELENBP1 and the adipocyte marker Adiponectin in two independent passages of wild-type cells before and after differentiation. (**C**) Representative immunoblot of SELENBP1 and adiponectin in stably transfected cells (shCtr and two independent shSELENBP1s, named sh85 and sh86), and corresponding densitometry analysis. β-Actin was used as loading control. D0: Day 0 (non-differentiated cells, i.e., pre-adipocytes); D7: Day 7 (mature (differentiated) adipocytes). DM: Differentiation Medium; MM: Maintenance Medium. ** *p* < 0.01 shows significant inhibition of SELENBP1 expression compared to control.

Standard protocols for 3T3-L1 differentiation into adipocyte-like cells require a mixture of insulin, dexamethasone, and 3-isobutyl-1-methylxanthine (IBMX) [30]. To avoid the use of the phosphodiesterase inhibitor IBMX (that would have masked the well-known [36,37] inhibitory effect of H_2_S on PDE itself), a modified differentiation protocol was developed (Figure 1A). Cells were seeded at 60% density and were grown until confluence for up to three days. On Day 0 (D0) cells were treated with differentiation medium and subsequently with maintenance medium on Days 2 and 4. Non-differentiated cells were processed for experiments on D0, while mature adipocytes were processed on Day 7 (D7). The efficiency of the newly designed protocol was assessed by Western blotting. Adiponectin expression (a known marker of differentiated adipocytes) was not detectable in non-differentiated cells, but it showed a strong induction upon differentiation, in line with previous studies [30]. Differences in adiponectin levels may simply depend on specific experimental conditions, including medium height, as previously reported [38]. Moreover, and in agreement with previous findings [39], the expression of SELENBP1 increased during the differentiation process (Figure 1B). To assess the role of SELENBP1 in the process of adipogenesis, we generated different stable SELENBP1 knock-down cell lines (designated as sh85 and sh86). The efficiency of the knock-down was assessed by Western blotting in adipocytes (Figure 1C). 

During differentiation, SELENBP1 was upregulated in shCtr (cells transfected with a non-coding shRNA construct), similarly to the results obtained in wild type cells. Both sh85 and sh86 displayed a pronounced knock-down efficiency. Adiponectin, a marker of early adipocyte differentiation, was induced on D7 shCtr and sh85 cells. However, sh86 cells displayed a strong reduction in adiponectin expression, indicating suppression of the differentiation process. These data show that among the shSELENBP1 constructs tested in this study, the sh86 line provides the more efficient SELENBP1 knock-down. Moreover, the sh86 cell line exhibits a suppression of adiponectin expression, predicting that SELENBP1 may act as an endogenous stimulator of the adipogenesis process.

### 3.2. SELENBP1 Knock-Down Suppresses Lipid Accumulation during Adipocyte Differentiation

**Figure 2:**
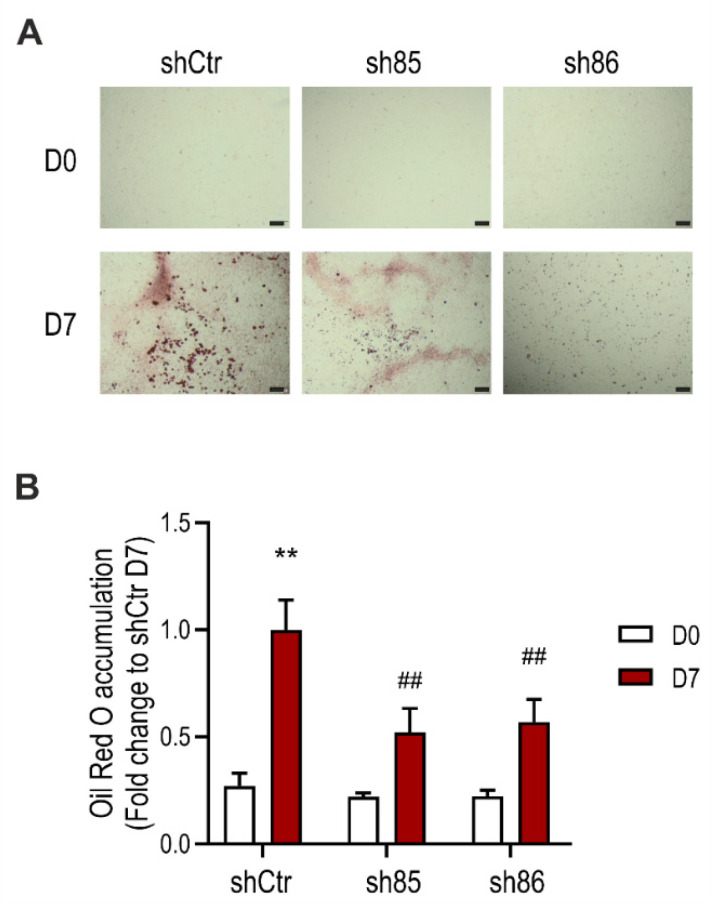
Effect of SELENBP1 knock-down on differentiation-associated lipid accumulation in 3T3-L1 cells. (**A**) Representative pictures of Oil Red O staining in shCtr and shSELENBP1 (sh85 and sh86 clones) cells before (Day 0) and after (Day 7) differentiation (scale bar: 200 µm). (**B**): numerical quantification of the findings. ** *p* < 0.01 indicates a significant difference between D7 and corresponding D0 values; ## *p* < 0.01 indicates a significant difference between shSELENBP1 (D7) and shCtr (D7).

Differentiation of 3T3-L1 fibroblasts into mature adipocytes is characterized by increased lipid production and growth arrest [40]. To determine the role of SELENBP1 knock-down on adipogenesis, we assessed lipid accumulation in shCtr and shSELENBP1 cells using the lipophilic dye Oil Red O. Fat deposits were increased upon differentiation, to a major extent in the control (shCtr) cells. On the other hand, lipid accumulation in the sh85 and sh86 cell lines was less compared to that of shCtr controls (Figure 2). These results suggest that SELENBP1 participates in the differentiation and associated lipid accumulation of 3T3-L1 cells.

### 3.3. Cellular H_2_S Levels Increase during Adipocyte Differentiation: Inhibitory Effect of SELENBP1 Knock-Down

**Figure 3:**
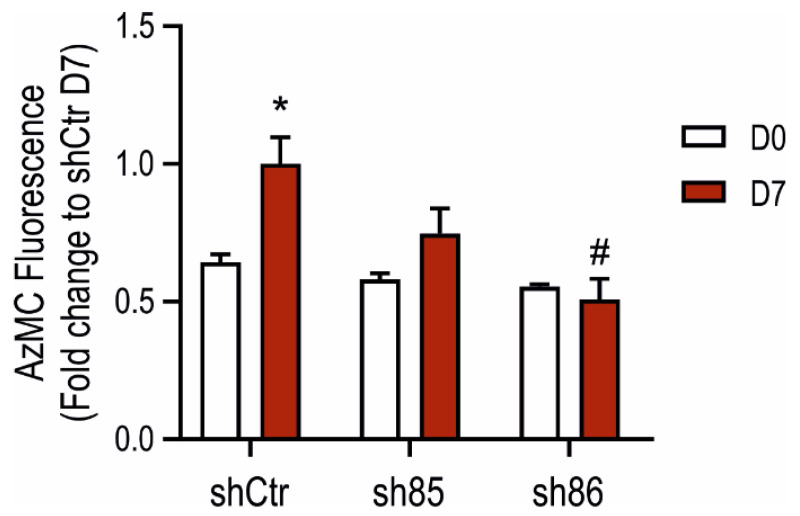
Increases in cellular H_2_S content during adipogenesis: role of SELENBP1. AzMC-assessed quantification of cellular H_2_S levels before and after differentiation in shCtr and shSELENBP1 cells (sh85, sh86 clones). * *p* < 0.05 shows a significant difference between D7 and the corresponding D0; # *p* < 0.05 shows a significant difference between shSELENBP1 (D7) and shCtr (D7).

H_2_S production in SELENBP1 knock-down cells was assessed using the AzMC assay. Upon differentiation, shCtr cell showed a pronounced increase in the H_2_S signal, while sh85 and sh86 did not display significant increases (Figure 3). Cellular H_2_S levels in sh86 cells were similarly low before vs. after differentiation (D0 vs. D7). These data indicate that cellular H_2_S levels increase during adipogenesis and suggest that SELENBP1 plays a role in the upregulation of H_2_S production during this process.

### 3.4. SELENBP1 Knock-Down Affects Cell Proliferation during Adipocyte Differentiation

**Figure 4:**
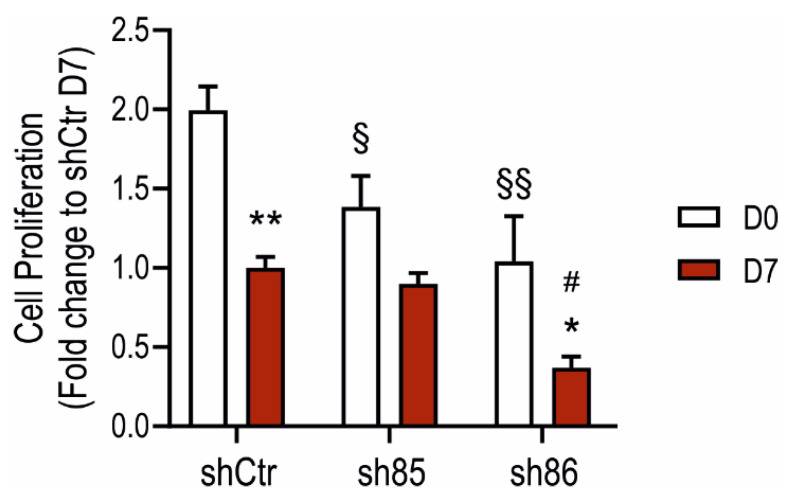
Effect of SELENBP1 silencing on adipocyte proliferation. Quantification of cell proliferation in shCtr and shSELENBP1 cells before and after differentiation. * *p* < 0.05 and ** *p* < 0.01 show significant differences between D7 and corresponding D0; # *p* < 0.05 shows a significant difference between shSELENBP1(D7) and shCtr(D7): ^§^ *p* < 0.05 and ^§§^ *p* < 0.01 show significant differences between shCtr (D0) and shSELENBP1(D0).

Next, we determined the effect of SELENBP1 knock-down on cell proliferation using the BrdU assay. All of the investigated cell lines showed an overall reduction in their proliferation rate during adipogenesis (Figure 4), in line with previous data [40]. At the undifferentiated stage, sh85 and sh86 displayed a significant reduction in cell growth compared to that of shCtr. Interestingly, differentiated shCtr and sh85 displayed comparable proliferation rates, which were similar to rates of the non-differentiated sh86 cells. These results suggests that SELENBP1 affects cell proliferation both in the undifferentiated cells (3T3-L1, D0) as well as in the mature adipocytes (D7).

### 3.5. Effect of SELENBP1 Knock-Down on Metabolic Activity

**Figure 5:**
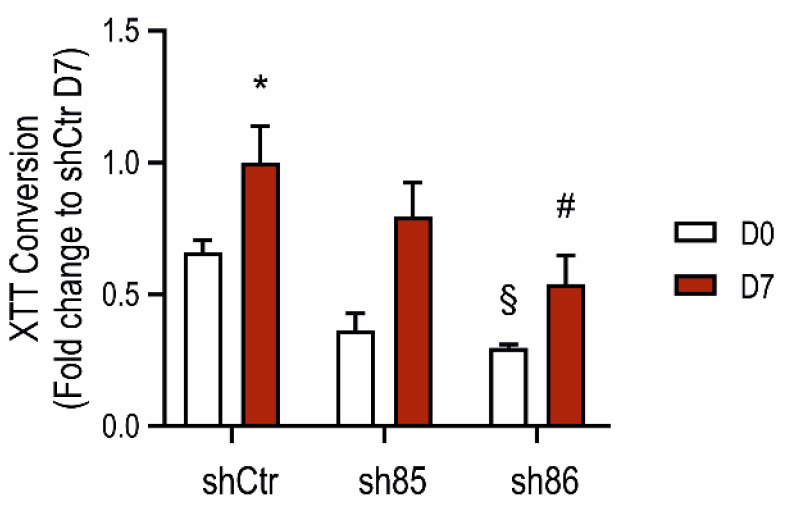
Effect of SELENBP1 silencing on the XTT-converting activity of adipocytes during differentiation. XTT conversion was quantified in control and SELENBP1 knock-down cells before and after differentiation. * *p* < 0.05 shows significant difference between D7 and corresponding D0; # *p* < 0.05 shows significant difference between shSELENBP1 (D7) and shCtr (D7); ^§^ *p* < 0.05 shows significant difference between shCtr (D0) and shSELENBP1 (D0).

The metabolic activity of control and SELENBP1 knock-down cells was measured by the XTT assay before and after differentiation. Sh86 cells displayed lower XTT-converting activity already at the non-differentiated stage compared to that of shCtr (Figure 5). This difference was also maintained during differentiation. Conversely, at D7, the XTT-converting activity of sh85 cells was similar to those of the control (shCtr) cells. These results suggest that SELENBP1 knock-down reduces the metabolic activity of both 3T3-L1 preadipocytes and mature adipocytes. 

### 3.6. SELENBP1 Knock-Down Decreases Mitochondrial Bioenergetics

**Figure 6:**
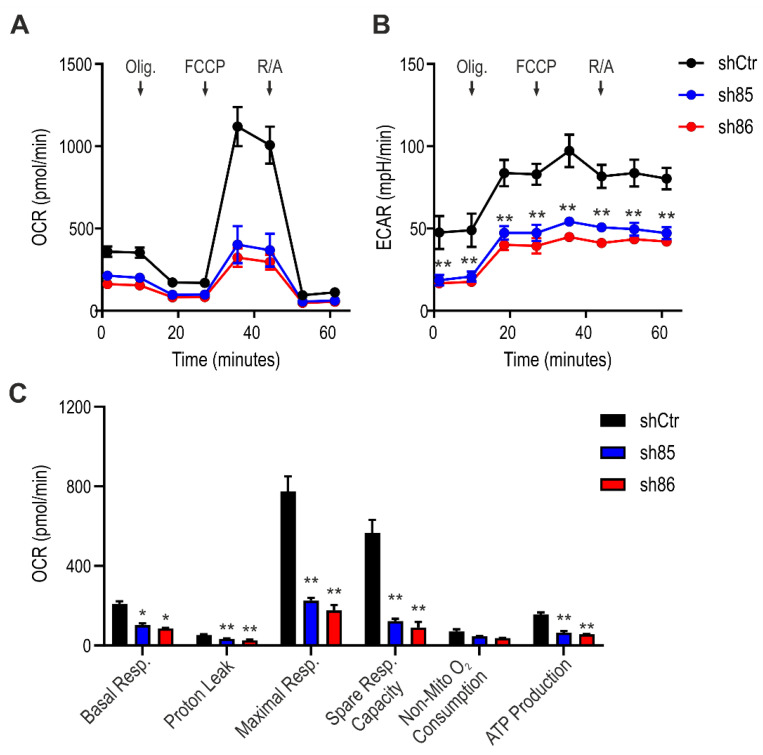
Effect of SELENBP1 silencing on mitochondrial respiration of mature adipocytes. (**A**) Oxygen consumption rate (OCR) and (**B**) extracellular acidification rate (ECAR) was measured in control and SELENBP1 knock-down cells after injection of oligomycin (Olig.), trifluoromethoxy carbonylcyanide phenylhydrazone (FCCP,) and Rotenone/antimycin A (AMA). (**C**) Analysis of different bioenergetics parameters obtained from OCR values. * *p* < 0.05 and ** *p* < 0.01 show significant differences between shSELENBP1 and shCtr.

To investigate the mitochondrial activity in control and SELENBP1 knock-down cells after differentiation, we performed an extracellular flux analysis. ShSELENBP1 cells displayed lower mitochondrial respiration compared to that of shCtr (Figure 6A), as well as lower glycolytic activity (Figure 6B) and an overall suppression of the bioenergetics parameters “basal respiration”, “maximal respiration”, “proton leak”, “spare respiratory capacity”, and “ATP production” (Figure 6C).

### 3.7. GYY4137 Modulates H_2_S-Producing Enzyme Expression in ShSELENBP1 Cells

**Figure 7:**
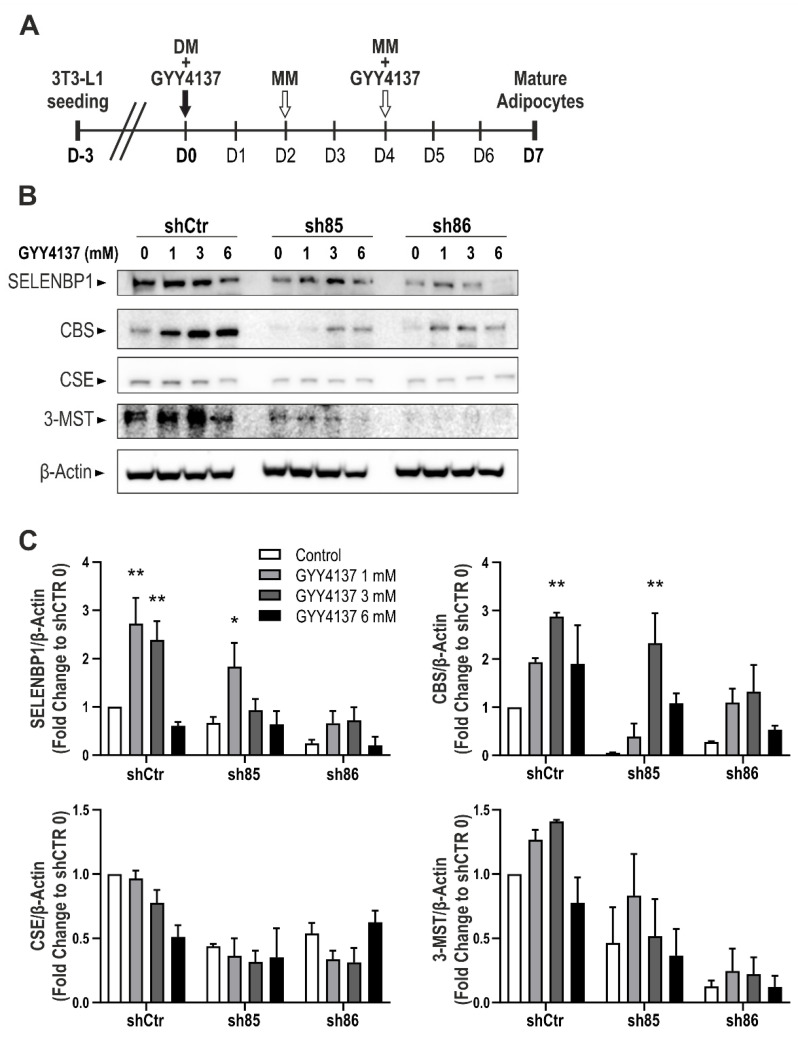
Effect of GYY4137 on H_2_S-producing enzymes. (**A**) Schematic representation of GYY4137 treatment during the differentiation process. (**B**) Representative immunoblots of SELENBP1, CBS, CSE, and 3-MST in shCtr and ShSELENBP1 cells (clones sh85 and sh86), differentiated in the presence of the H_2_S donor GYY4137. (**C**) Quantification of the densitometric data. * *p* < 0.05 and ** *p* < 0.01 show significant differences between GYY4137-treated cells and the corresponding control.

The expression of CBS, CSE, 3-MST, and SELENBP1 increase in a time-dependent manner in 3T3-L1 cells subjected to differentiation [39,41]. Here, we tested the effect of GYY4137 and SELENBP1 knock-down cells on the expression of the H_2_S-sytnthetizing enzymes. The H_2_S donor was administered twice during the differentiation process (D0 and D4) (Figure 7A). Subsequently, we investigated the effect of GYY4137 on the expression of SELENBP1 and the three main H_2_S producing enzymes: CBS, CSE, and 3-MST (Figure 7A,B). Treatment with GYY4137 modulated the expression of Selenbp1, CBS, and 3-MST in control cells (shCtr) and sh85, following a bell-shaped model (upregulation with 1–3 mM, downregulation at 6 mM). Interestingly, GYY4137 administration in sh86 cells upregulated the expression of SELENBP1 and CBS to a level comparable to that of control cells (schCtr 0). Interestingly, in untreated cells, the expression of CBS, CSE, and 3-MST was downregulated in response to SELENBP1 silencing, suggesting that SELENBP1 regulates the expression of the H_2_S-producing enzymes during adipogenesis. The finding that the H_2_S donor GYY4137 restored this downregulation, however, indicates that the regulation of these enzymes may be the transcriptional consequence of alterations in cellular H_2_S levels. 

### 3.8. H_2_S Donation Restores Lipid Accumulation in SELENBP1 Knock-Down Adipocytes

**Figure 8:**
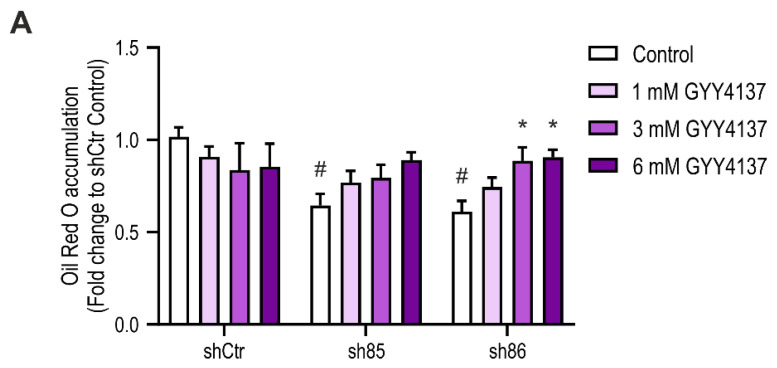
Effect of the H_2_S donor GYY4137 on differentiation-associated lipid accumulation in shSELENBP1 knock-down cells. (**A**) quantification of Oil Red O staining in shCtr and shSELENBP1 (sh85, sh86 clones) treated with 1, 3, and 6 mM GYY4137. * *p* < 0.05 indicates a significant difference between GYY4137-treated and corresponding untreated values; # *p* < 0.05 indicates a significant difference between shSELENBP1 (untreated) and shCtr (untreated).

Next, we tested the effect of the H_2_S-releasing molecule GYY4137 on SELENBP1 knock-down cells. The H_2_S donor was administered twice during the differentiation process (D0 and D4) (Figure 8A). The effect of 1, 3, and 6 mM GYY4137 on SELENBP1 knock-down cells was then evaluated by Oil Red O staining after differentiation (D7). Treatment with the H_2_S donor significantly increased fat deposits in shSELENBP1 cells; at 6 mM of the H_2_S donor, lipid accumulation was similar to the accumulation measured in control cells (Figure 8B). These data further indicate that H_2_S produced by SELENBP1 during the differentiation process is the mediator involved in lipid accumulation during adipogenesis.

### 3.9. Effect of GYY4137 on Cellular H_2_S Levels in SELENBP1 Knock-Down Adipocytes

**Figure 9:**
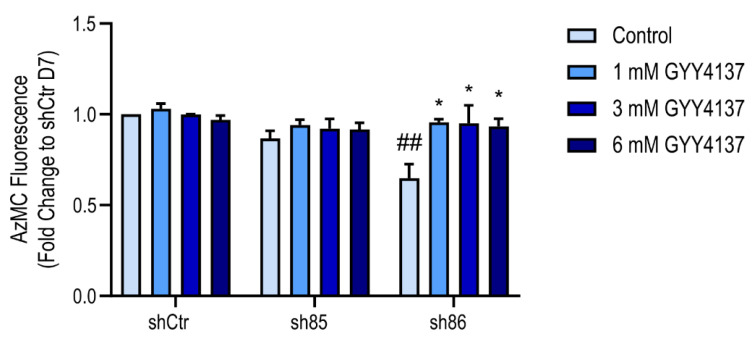
Increased cellular H_2_S content in SELENBP1 knock-down cells treated with GYY4137. AzMC-assessed quantification of cellular H_2_S levels in shCtr and shSELENBP1 cells (sh85, sh86 clones) treated with GYY4137 during the differentiation process. * *p* < 0.05 shows a significant difference between GYY4137-treated cells and the group control; ## *p* < 0.01 indicates a significant difference between shSELENBP1 (untreated) and shCtr (untreated).

H_2_S production in SELENBP1 knock-down cells differentiated in the presence of GYY4137 was assessed using the AzMC assay. Treatment with GYY4137 significantly increased the H_2_S signal in sh86 cells (Figure 9), while cellular H_2_S levels in shCtr and sh85 cells were similar to the corresponding untreated value. As shown earlier in Figure 8, GYY4137 also upregulated the expression of various H_2_S-producing enzymes in the shSELENBP1 cells. These data indicate that treatment with GYY4137 is able to increase H_2_S levels in SELENBP1 knock-down cells via direct and indirect mechanisms. 

### 3.10. GYY4137 Improves Bioenergetics in SELENBP1 Knock-Down Adipocytes

**Figure 10:**
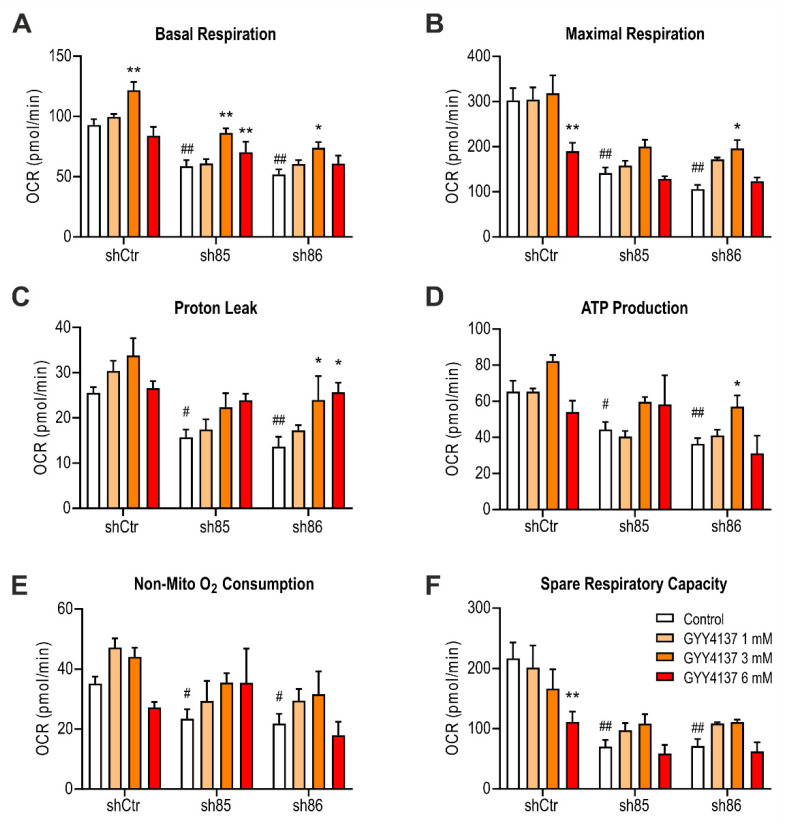
Effect of GYY4137 on bioenergetics parameters in SELENBP1 knock-down cells. (**A**) Basal respiration, (**B**) maximal respiration, (**C**) proton leak, (**D**) ATP production, (**E**) non-mitochondrial oxygen consumption, and (**F**) spare respiratory capacity were calculated after extracellular flux analysis on GYY4137-treated shSELENBP1 cells. ** *p* < 0.01 shows a significant difference between GYY4137-treated cells and the group control; # *p* < 0.05 and ## *p* < 0.01 indicate significant differences between shSELENBP1 (untreated) and shCtr (untreated).

To investigate the effect of GYY4137 treatment on mitochondrial respiration, we performed extracellular flux analysis on shSELENBP1 cells. Treatment with 1 and 3 mM GYY4137 partially restored bioenergetic parameters in SELENBP1 knock-down cells. The highest concentration of the H_2_S donor (6 mM) was no longer effective in enhancing mitochondrial function in SELENBP1 knock-down cells; this concentration of the donor also significantly decreased the maximal respiration (Figure 10B) and spare respiratory capacity (Figure 10F) of shCtr cells. These pharmacological effects are consistent with the well-known [17] biphasic or bell-shaped concentration-response of H_2_S on mitochondrial activity. 

## 4. Discussion

The findings presented in the current report can be summarized as follows: (1) Adipocyte differentiation (lipid accumulation) is supported by SELENBP1 because this process is suppressed by stable SELENBP1 silencing. (2) Stable SELENBP1 silencing also suppresses cell proliferation and metabolic activity during differentiation. (3) Adipocyte differentiation is associated with an upregulation of H_2_S biosynthesis. (4) H_2_S donation using the slow-release donor GYY4137 partially restores the phenotype of shSELENBP1 cells in terms of lipid accumulation, cellular H_2_S levels, and cellular bioenergetics. (5) H_2_S biosynthesis during adipocyte differentiation is, at least in part, the consequence of SELENBP1 activity, because SELENBP1-silencing decreases the H_2_S signal during differentiation. These conclusions are made through the comparison of sham-silenced cells vs. the shSELENBP1 (sh86 clone, i.e., the clone exhibiting the most efficient SELENBP1 silencing). Although, traditionally, CBS, CSE, and 3-MST are considered the major H_2_S-producing enzymes, the current results indicate that SELENBP1 also contributes, at least in the current experimental system (adipocytes), and its expression correlates with the expression of the three “traditional” H_2_S-producing enzymes (CBS, CSE, and 3-MST). SELENBP1 expression and cellular H_2_S levels are lower (although not undetectable) in pre-adipocytes. Indeed, previous work has already demonstrated that CBS, CSE, and 3-MST are also present in 3T3-L1 cells, and their expression (as well as cellular H_2_S levels) is increased during the differentiation of these cells into adipocytes [40]. In fact, in these prior studies, silencing or pharmacological inhibition of CBS, CSE, or 3-MST (similar to the silencing of SELENBP1 in the current experiments) suppressed the accumulation of lipids during adipocyte differentiation [40]. These data, as well as additional experiments focusing solely on CSE [41,42,43], suggest that a global upregulation of H_2_S biosynthesis constitutes a significant component of the process of adipogenesis. The molecular mechanisms involved in the upregulation of the various H_2_S-producing enzymes during adipogenesis remain to be characterized in future studies. Similarly, the molecular mechanisms by which the increased intracellular H_2_S stimulates fat accumulation remain to be further characterized; one potential pathway involves PPARγ transactivation (activation of PPARγ being a well-established pathway that promotes adipogenesis); H_2_S was shown to activate this pathway by S-sulfhydrating the cysteine residues in its DNA binding domain [41,42].

If, indeed, endogenous H_2_S (produced by CBS, CSE, 3-MST, SELENBP1, or possibly additional enzymatic or non-enzymatic sources) acts a stimulator of adipogenesis and cellular fat accumulation, then one would expect to find elevated H_2_S levels in adipose subjects, and a reduction of fat accumulation in animals treated with pharmacological inhibitors of H_2_S biosynthesis. There are, in fact, several reports showing that obesity is associated with significant elevations in exhaled and plasma H_2_S levels in human subjects [44,45]. In addition, recent data show that CSE^-/-^ mice placed on a high-fat diet develop less white fat accumulation than do the corresponding wild-type controls [46]. In addition, heterozygous deletion of the Mpst gene (encoding for 3-MST) was found to ameliorate hepatic steatosis in high fat diet-fed mice [47]. Moreover, supplementation of H_2_S during a high fat diet accelerates white fat accumulation in wild-type mice [46]. All of these data reinforce the concept that H_2_S (endogenously produced, or even exogenously administered) acts as a pro-adipogenic factor. However, there are other sets of data that are in disagreement with the above concept: H_2_S-producing enzymes were found to be decreased in adipose tissues in murine models of obesity [48,49]. It is important to emphasize that H_2_S regulates a multitude of processes that can influence cellular energy expenditure, metabolism, and cellular lipid accumulation. These processes include insulin secretion, insulin resistance, carbohydrate metabolism, mitochondrial function, beta-oxidation, and lipolysis [50,51,52,53,54,55,56,57,58,59,60]; the complex interaction between these processes in various stages of the adipogenesis process and the individual or composite role of the various H_2_S-producing enzymes remain to be further investigated in future studies. Importantly, the amount of cellular lipids is determined by a balance of lipid accumulation and lipolysis. Interestingly, Ding and colleagues have recently implicated H_2_S (produced by CSE) in the regulation of lipolysis: H_2_S was found to exert an inhibitory effect on this process; the mechanism was attributed to the posttranscriptional regulation (sulfhydration) of perilipin 1 (a well-established regulator of lipid-droplet formation and triglyceride storage) followed by the stabilization of peripilin-1 distribution, which, in turn, blocks the translocation of hormone sensitive lipase (HSL) to lipid droplets, with the net result being an inhibition of lipolysis and an increase in fat mass [46].

Although in some of the assays used in the current study the effects of SELENBP1 silencing on mitochondrial activity and on adipocyte differentiation corresponded to each other, and all forms of cell differentiation and adipogenesis are energy-demanding processes, it would be an oversimplification to claim that the mechanism by which endogenous, SELENBP1-derived H_2_S contributes to adipogenesis is through increasing cellular energy production. For instance, and in line with the bell-shaped role of H_2_S in the modulation of mitochondrial function [17], the highest concentration of the H_2_S donor used in the current study (6 mM GYY4137) was no longer stimulatory on mitochondrial function, but this concentration of the donor (similar to the lower concentrations used, which increased mitochondrial respiration) simulated adipogenesis in SELENBP1-silenced cells. It is likely that H_2_S exerts multiple biological effects in the differentiating adipocyte (on multiple cellular signaling processes as well as on cellular bioenergetic function), and the net result observed is a combination of these actions. (The multiple molecular mechanisms by which lower concentrations of H_2_S stimulate, while higher concentrations of H_2_S suppress, mitochondrial function in health and disease have recently been reviewed [17,19,51,61].) 

Taken together, the current results suggest SELENBP1 is an endogenous stimulator of adipocyte maturation, adipogenesis, and cellular lipid accumulation. In SELENBP1 cells, H_2_S levels decreased, although in the current experiments we did not supplement our cells with exogenous methanethiol. However, the cells may contain relevant concentrations of methanethiol (as a substrate of SELENBP1 for H_2_S generation), because methanethiol can be generated by mammalian cells from methionine [62,63]. Thus, one possible mechanism for the lowered H_2_S levels in shSELENBP1 cells may be a direct mechanism (reduced conversion of methanethiol to H_2_S in the absence of the converting enzyme). Another, indirect mechanism may be related to the fact that in shSELENBP1 cells, CBS, CSE, and 3-MST expression was suppressed (Figure 7). Thus, SELENBP1 appears to regulate the levels of these “traditional” H_2_S-producing systems, which may have also contributed to the lowering of H_2_S levels in the SELENBP1-silenced cells. Indeed, the ability of SELENBP1 to modulate various signal transduction pathways and gene expression patterns has been previously documented in various cell types in various contexts [28,64,65,66].

The other enzymatic product of SELENBP1, H_2_O_2_ (which is produced in equimolar amounts with H_2_S during the process, whereby SELENBP1 catalyzes the conversion of methanethiol, water, and oxygen to formaldehyde), should also be mentioned in the context of adipogenesis. H_2_O_2_ may, in fact, also play a role in the stimulation of adipogenesis; studies with catalase inhibition or catalase silencing have implicated an independent role of cellular H_2_O_2_ production in the stimulation of adipocyte hyperplasia and hypertrophy [67,68]. Although the ability of SELENBP1 to regulate H_2_S levels in mammalian cells and tissues is likely ([28,29] and the present study), the amount of available information on the role of the SELENBP1 pathway in the regulation of various cell functions is minimal and remains to be further explored in future studies.

## 5. Conclusions

We conclude that SELENBP1 in adipocytes (1) contributes to H_2_S biosynthesis and (2) acts as an endogenous stimulator of adipocyte differentiation.

## Data Availability

Data available on request.

## References

[1] Szabo C. (2018). A timeline of hydrogen sulfide (H_2_S) research: From environmental toxin to biological mediator. Biochem. Pharmacol..

[2] Aroca A., Gotor C., Bassham D.C., Romero L.C. (2020). Hydrogen sulfide: From a toxic molecule to a key molecule of cell life. Antioxidants.

[3] Kimura H. (2002). Hydrogen sulfide as a neuromodulator. Mol. Neurobiol..

[4] Szabo C. (2007). Hydrogen sulphide and its therapeutic potential. Nat. Rev. Drug Discov..

[5] Kimura H. (2011). Hydrogen sulfide: Its production, release and functions. Amino Acids.

[6] Li L., Rose P., Moore P.K. (2011). Hydrogen sulfide and cell signaling. Annu. Rev. Pharmacol. Toxicol..

[7] Whiteman M., Winyard P.G. (2011). Hydrogen sulfide and inflammation: The good, the bad, the ugly and the promising. Expert Rev. Clin. Pharmacol..

[8] Predmore B.L., Lefer D.J., Gojon G. (2012). Hydrogen sulfide in biochemistry and medicine. Antioxid. Redox. Signal..

[9] Wang R. (2012). Physiological implications of hydrogen sulfide: A whiff exploration that blossomed. Physiol. Rev..

[10] Li Q., Lancaster J.R. (2013). Chemical foundations of hydrogen sulfide biology. Nitric Oxide.

[11] Kimura H. (2014). The physiological role of hydrogen sulfide and beyond. Nitric Oxide.

[12] Yang G., Wang R. (2015). H_2_S and blood vessels: An overview. Handbook of Experimental Pharmacolology.

[13] Wang R., Szabo C., Ichinose F., Ahmed A., Whiteman M., Papapetropoulos A. (2015). The role of H_2_S bioavailability in endothelial dysfunction. Trends Pharmacol. Sci..

[14] Papapetropoulos A., Whiteman M., Cirino G. (2015). Pharmacological tools for hydrogen sulphide research: A brief, introductory guide for beginners. Br. J. Pharmacol..

[15] Szabo C. (2017). Hydrogen sulfide, an enhancer of vascular nitric oxide signaling: Mechanisms and implications. Am. J. Physiol. Cell. Physiol..

[16] Szabo C., Papapetropoulos A. (2017). International Union of Basic and Clinical Pharmacology. CII: Pharmacological Modulation of H_2_S Levels: H_2_S Donors and H_2_S Biosynthesis Inhibitors. Pharmacol. Rev..

[17] Blachier F., Andriamihaja M., Larraufie P., Ahn E., Lan A., Kim E. (2020). Production of hydrogen sulfide by the intestinal microbiota and epithelial cells and consequences for the colonic and rectal mucosa. Am. J. Physiol. Gastrointest. Liver Physiol..

[18] Szabo C. (2020). The re-emerging pathophysiological role of the cystathionine-beta-synthase—Hydrogen sulfide system in Down syndrome. FEBS J..

[19] Dilek N., Papapetropoulos A., Toliver-Kinsky T., Szabo C. (2020). Hydrogen sulfide: An endogenous regulator of the immune system. Pharmacol. Res..

[20] Zuhra K., Augsburger F., Majtan T., Szabo C. (2020). Cystathionine-β-synthase: Molecular regulation and pharmacological inhibition. Biomolecules.

[21] Cortese-Krott M.M., Koning A., Kuhnle G.G.C., Nagy P., Bianco C.L., Pasch A., Wink D.A., Fukuto J.M., Jackson A.A., van Goor H. (2017). The reactive species interactome: Evolutionary emergence, biological significance, and opportunities for redox metabolomics and personalized medicine. Antioxid. Redox. Signal..

[22] Kimura H. (2019). Signaling by hydrogen sulfide (H_2_S) and polysulfides (H_2_Sn) in the central nervous system. Neurochem. Int..

[23] Sies H., Jones D.P. (2020). Reactive oxygen species (ROS) as pleiotropic physiological signalling agents. Nat. Rev. Mol. Cell. Biol..

[24] Shibuya N., Koike S., Tanaka M., Ishigami-Yuasa M., Kimura Y., Ogasawara Y., Fukui K., Nagahara N., Kimura H. (2013). A novel pathway for the production of hydrogen sulfide from D-cysteine in mammalian cells. Nat. Commun..

[25] Souza L.K., Araújo T.S., Sousa N.A., Sousa F.B., Nogueira K.M., Nicolau L.A., Medeiros J.V. (2017). Evidence that d-cysteine protects mice from gastric damage via hydrogen sulfide produced by d-amino acid oxidase. Nitric Oxide.

[26] Akaike T., Ida T., Wei F.Y., Nishida M., Kumagai Y., Alam M.M., Ihara H., Sawa T., Matsunaga T., Kasamatsu S. (2017). Cysteinyl-tRNA synthetase governs cysteine polysulfidation and mitochondrial bioenergetics. Nat. Commun..

[27] Pol A., Renkema G.H., Tangerman A., Winkel E.G., Engelke U.F., de Brouwer A.P.M., Lloyd K.C., Araiza R.S., van den Heuvel L., Omran H. (2018). Mutations in SELENBP1, encoding a novel human methanethiol oxidase, cause extraoral halitosis. Nat. Genet..

[28] Elhodaky M., Hong L.K., Kadkol S., Diamond A.M. (2020). Selenium-binding protein 1 alters energy metabolism in prostate cancer cells. Prostate.

[29] Tangerman A. (2009). Measurement and biological significance of the volatile sulfur compounds hydrogen sulfide, methanethiol and dimethyl sulfide in various biological matrices. J. Chromatogr. B Anal. Technol. Biomed. Life Sci..

[30] Zebisch K., Voigt V., Wabitsch M., Brandsch M. (2012). Protocol for effective differentiation of 3T3-L1 cells to adipocytes. Anal. Biochem..

[31] Randi E.B., Vervaet B., Tsachaki M., Porto E., Vermeylen S., Lindenmeyer M.T., Thuy L.T.T., Cohen C.D., Devuyst O., Kistler A.D. (2020). The antioxidative role of cytoglobin in podocytes: Implications for a role in chronic kidney disease. Antioxid. Redox. Signal..

[32] Augsburger F., Randi E.B., Jendly M., Ascencao K., Dilek N., Szabo C. (2020). Role of 3-mercaptopyruvate sulfurtransferase in the regulation of proliferation, migration, and bioenergetics in murine colon cancer cells. Biomolecules.

[33] Ascenção K., Dilek N., Augsburger F., Panagaki T., Zuhra K., Szabo C. (2021). Pharmacological induction of mesenchymal-epithelial transition via inhibition of H_2_S biosynthesis and consequent suppression of ACLY activity in colon cancer cells. Pharmacol. Res..

[34] Szczesny B., Módis K., Yanagi K., Coletta C., Le Trionnaire S., Perry A., Wood M.E., Whiteman M., Szabo C. (2014). AP39, a novel mitochondria-targeted hydrogen sulfide donor, stimulates cellular bioenergetics, exerts cytoprotective effects and protects against the loss of mitochondrial DNA integrity in oxidatively stressed endothelial cells in vitro. Nitric Oxide.

[35] Panagaki T., Randi E.B., Augsburger F., Szabo C. (2019). Overproduction of H_2_S, generated by CBS, inhibits mitochondrial Complex IV and suppresses oxidative phosphorylation in Down syndrome. Proc. Natl. Acad. Sci. USA.

[36] Bucci M., Papapetropoulos A., Vellecco V., Zhou Z., Pyriochou A., Roussos C., Roviezzo F., Brancaleone V., Cirino G. (2010). Hydrogen sulfide is an endogenous inhibitor of phosphodiesterase activity. Arterioscler. Thromb. Vasc. Biol..

[37] Bucci M., Papapetropoulos A., Vellecco V., Zhou Z., Zaid A., Giannogonas P., Cantalupo A., Dhayade S., Karalis K.P., Wang R. (2012). cGMP-dependent protein kinase contributes to hydrogen sulfide-stimulated vasorelaxation. PLoS ONE.

[38] Sheng X., Tucci J., Malvar J., Mittelman S.D. (2014). Adipocyte differentiation is affected by media height above the cell layer. Int J. Obes..

[39] Steinbrenner H., Micoogullari M., Hoang N.A., Bergheim I., Klotz L.O., Sies H. (2019). Selenium-binding protein 1 (SELENBP1) is a marker of mature adipocytes. Redox. Biol..

[40] Green H., Meuth M. (1974). An established pre-adipose cell line and its differentiation in culture. Cell.

[41] Tsai C.Y., Peh M.T., Feng W., Dymock B.W., Moore P.K. (2015). Hydrogen sulfide promotes adipogenesis in 3T3L1 cells. PLoS ONE.

[42] Cai J., Shi X., Wang H., Fan J., Feng Y., Lin X., Yang J., Cui Q., Tang C., Xu G. (2016). Cystathionine γ lyase-hydrogen sulfide increases peroxisome proliferator-activated receptor γ activity by sulfhydration at C139 site thereby promoting glucose uptake and lipid storage in adipocytes. Biochim. Biophys. Acta Mol. Cell Biol..

[43] Yang G., Ju Y., Fu M., Zhang Y., Pei Y., Racine M., Baath S., Merritt T.J.S., Wang R., Wu L. (2018). Cystathionine gamma-lyase/hydrogen sulfide system is essential for adipogenesis and fat mass accumulation in mice. Biochim. Biophys. Acta Mol. Cell Biol. Lipids.

[44] Alkhouri N., Eng K., Cikach F., Patel N., Yan C., Brindle A., Rome E., Hanouneh I., Grove D., Lopez R. (2015). Breathprints of childhood obesity: Changes in volatile organic compounds in obese children compared with lean controls. Pediatr. Obes..

[45] Comas F., Latorre J., Ortega F., Arnoriaga Rodríguez M., Lluch A., Sabater M., Rius F., Ribas X., Costas M., Ricart W. (2020). Morbidly obese subjects show increased serum sulfide in proportion to fat mass. Int. J. Obes..

[46] Ding Y., Wang H., Geng B., Xu G. (2020). Sulfhydration of perilipin 1 is involved in the inhibitory effects of cystathionine gamma lyase/hydrogen sulfide on adipocyte lipolysis. Biochem. Biophys. Res. Commun..

[47] Li M., Xu C., Shi J., Ding J., Wan X., Chen D., Gao J., Li C., Zhang J., Lin Y. (2018). Fatty acids promote fatty liver disease via the dysregulation of 3-mercaptopyruvate sulfurtransferase/hydrogen sulfide pathway. Gut.

[48] Peh M.T., Anwar A.B., Ng D.S., Atan M.S., Kumar S.D., Moore P.K. (2014). Effect of feeding a high fat diet on hydrogen sulfide (H_2_S) metabolism in the mouse. Nitric Oxide.

[49] Katsouda A., Szabo C., Papapetropoulos A. (2018). Reduced adipose tissue H_2_S in obesity. Pharmacol. Res..

[50] Feng X., Chen Y., Zhao J., Tang C., Jiang Z., Geng B. (2009). Hydrogen sulfide from adipose tissue is a novel insulin resistance regulator. Biochem. Biophys. Res. Commun..

[51] Whiteman M., Gooding K.M., Whatmore J.L., Ball C.I., Mawson D., Skinner K., Tooke J.E., Shore A.C. (2010). Adiposity is a major determinant of plasma levels of the novel vasodilator hydrogen sulphide. Diabetologia.

[52] Szabo C., Ransy C., Módis K., Andriamihaja M., Murghes B., Coletta C., Olah G., Yanagi K., Bouillaud F. (2014). Regulation of mitochondrial bioenergetic function by hydrogen sulfide. Part I. Biochemical and physiological mechanisms. Br. J. Pharmacol..

[53] Carter R.N., Morton N.M. (2016). Cysteine and hydrogen sulphide in the regulation of metabolism: Insights from genetics and pharmacology. J. Pathol..

[54] Bełtowski J., Jamroz-Wiśniewska A. (2016). Hydrogen sulfide in the adipose tissue-physiology, pathology and a target for pharmacotherapy. Molecules.

[55] Katsouda A., Bibli S.I., Pyriochou A., Szabo C., Papapetropoulos A. (2016). Regulation and role of endogenously produced hydrogen sulfide in angiogenesis. Pharmacol. Res..

[56] Pichette J., Gagnon J. (2016). Implications of hydrogen sulfide in glucose regulation: How H_2_S can alter glucose homeostasis through metabolic hormones. Oxid. Med. Cell. Longev..

[57] Candela J., Velmurugan G.V., White C. (2016). Hydrogen sulfide depletion contributes to microvascular remodeling in obesity. Am. J. Physiol. Heart Circ. Physiol..

[58] Zhang H., Huang Y., Chen S., Tang C., Wang G., Du J., Jin H. (2020). Hydrogen sulfide regulates insulin secretion and insulin resistance in diabetes mellitus, a new promising target for diabetes mellitus treatment? A review. J. Adv. Res..

[59] Ali A., Wang Y., Wu L., Yang G. (2020). Gasotransmitter signaling in energy homeostasis and metabolic disorders. Free Radic. Res..

[60] Gheibi S., Samsonov A.P., Gheibi S., Vazquez A.B., Kashfi K. (2020). Regulation of carbohydrate metabolism by nitric oxide and hydrogen sulfide: Implications in diabetes. Biochem. Pharmacol..

[61] Szabo C. (2021). Hydrogen sulfide, an endogenous stimulator of mitochondrial function in cancer cells. Cells.

[62] Scislowski P.W., Pickard K. (1994). The regulation of transaminative flux of methionine in rat liver mitochondria. Arch. Biochem. Biophys..

[63] Yamada H., Akahoshi N., Kamata S., Hagiya Y., Hishiki T., Nagahata Y., Matsuura T., Takano N., Mori M., Ishizaki Y. (2012). Methionine excess in diet induces acute lethal hepatitis in mice lacking cystathionine γ-lyase, an animal model of cystathioninuria. Free Radic. Biol. Med..

[64] Zhao C., Zeng H., Wu R.T., Cheng W.H. (2016). Loss of selenium-binding protein 1 decreases sensitivity to clastogens and intracellular selenium content in HeLa cells. PLoS ONE.

[65] Caswell D.R., Chuang C.H., Ma R.K., Winters I.P., Snyder E.L., Winslow M.M. (2018). Tumor suppressor activity of Selenbp1, a direct Nkx2-1 target, in lung adenocarcinoma. Mol. Cancer Res..

[66] Wang Y., Zhu W., Chen X., Wei G., Jiang G., Zhang G. (2020). Selenium-binding protein 1 transcriptionally activates p21 expression via p53-independent mechanism and its frequent reduction associates with poor prognosis in bladder cancer. J. Transl. Med..

[67] Shin S.K., Cho H.W., Song S.E., Im S.S., Bae J.H., Song D.K. (2020). Oxidative stress resulting from the removal of endogenous catalase induces obesity by promoting hyperplasia and hypertrophy of white adipocytes. Redox. Biol..

[68] Nitta Y., Muraoka-Hirayama S., Sakurai K. (2020). Catalase is required for peroxisome maintenance during adipogenesis. Biochim. Biophys. Acta Mol. Cell Biol. Lipids.

